# Urban health extension program model housing and household visits improved the utilization of health Services in Urban Ethiopia: a community-based cross-sectional study

**DOI:** 10.1186/s12913-019-3868-9

**Published:** 2019-01-14

**Authors:** Nebiyou Tafesse, Aregawi Gesessew, Ergataw Kidane

**Affiliations:** 1 Menelik II Health Science College, Kotebe Metropolitan University, PO Box: 3268, Addis Ababa, Ethiopia; 2 Yekatit 12 Hospital Medical College, Addis Ababa Health Bureau, Addis Ababa, Ethiopia

**Keywords:** UHE-ps, Home visit, Model household, Graduated, Health services, Non-model household

## Abstract

**Background:**

The government of Ethiopia launched an innovative program called Urban Health Extension Program (UHEP) in 2009, aims to produce better health outcomes to the urban populations using urban health extension professionals (UHE-ps) by enabling households to implement most health intervention packages designed by the government, which is referred to as model households (practice and implement at least 75% of the training provided by UHE-ps on UHEP packages). The objective of this study was to assess health service use and its associated factors.

**Methods:**

A community-based cross-sectional study was conducted to assess the health service use in Addis Ababa. Structured questionnaires were filled out by 1086 women, and a binary logistic regression was performed.

**Results:**

Urban health extension professionals performed home visits to 57.1% (95% CI (confidence interval) =54.2 to 59.8%) of the households. Mothers who had heard of the program (had information about UHEP) were 2.13 times more likely to visit the health center (HC) (AOR (adjusted odds ratio) =2.13, 95% CI = 1.36 to 3.32) than mothers who had not heard of the program. Mothers from model households were 2.12 times more likely to visit the HC (AOR = 2.12, 95% CI = 1.16 to 3.88) than mothers from non-model households. Mothers whose households were visited by the UHE-ps were 1.89 times more likely to visit the HC (AOR = 1.89, 95% CI = 1.22 to 2.94) than mothers whose households were not visited. Similarly, mothers who were in the reproductive age group (18 to 49 years) were 1.74 times more likely to visit the HC (AOR = 1.74, 95%CI = 1.12 to 2.71) than mothers above 49 years old.

**Conclusions:**

Model households and mothers in the reproductive age group exhibited significant associations with health service use. Sustaining the practices of graduated and certified model households is essential to maximize the benefits of the UHEP’s activities regarding health service use. Regular home visits to both model and non-model households are essential to scale up health service use and design re-graduation or other sustainable options for already graduated households.

## Background

The government of Ethiopia launched an innovative program called Urban Health Extension Program (UHEP) in 2009, intended to produce good health status to the urban populations using urban health extension professionals (UHE-ps)as lead implementer of the program [1]. The purpose of the program is to ensure access and quality of services for the urban community. With 15 UHEP packages, the UHEP was initiated in seven regions of the country (Tigray, Amhara, Oromia, SNNPR (southern nation, nationalities people’s region) Harari, DD (Diredawa) and (AA) (Addis Ababa) [2]. The site of work for the (UHE-ps) is in the health centers serving 40, 000 community members.

The ultimate aim of the UHEP is to produce model households. The health service extension program contains 16 rural extension program packages, since there is separate health education and communication package (in contrast to the UHEP packages, of which there are 15 because it is thought that this package can serve as a tool for all the packages of the UHEP (Table 1). Households that attend at least 75% of the training and put at least 75% of the UHEP packages into practice will be graduated and certified by the catchment UHE-ps [2].

**Table 1:** The 15 UHEP packages implemented in Addis Ababa, Ethiopia, 2015

s.no	Type of packages	Packages of the UHEP
1	Disease prevention and control	1. HIV/AIDS and tuberculosis control
		2. Malaria prevention and control
		3. First aid and emergency measures
2	Family health	1. Maternal and child health
		2. Family planning
		3. Immunization
		4. Nutrition
		5. Adolescent reproductive health
3	Hygiene and environmental sanitation	1. Excreta disposal
		2. Solid and liquid waste disposal
		3. Water supply and safety measures
		4. Food hygiene and safety measures
		5. Healthy housing
		6. Control of insects and rodents
		7. Personal hygiene

A number of factors have been identified as the leading causes of poor health service utilization such as inaccessibility of the health care facility, low socioeconomic status, low educational status of the mothers and cultural views and insights of the community. A number of studies showed that these factors categorized as sociodemographic status, autonomy of women’s, cultural insights, trends of diseases and health service utilizations, and physical and economic accessibility issues [3–5].

**Fig. 1:**
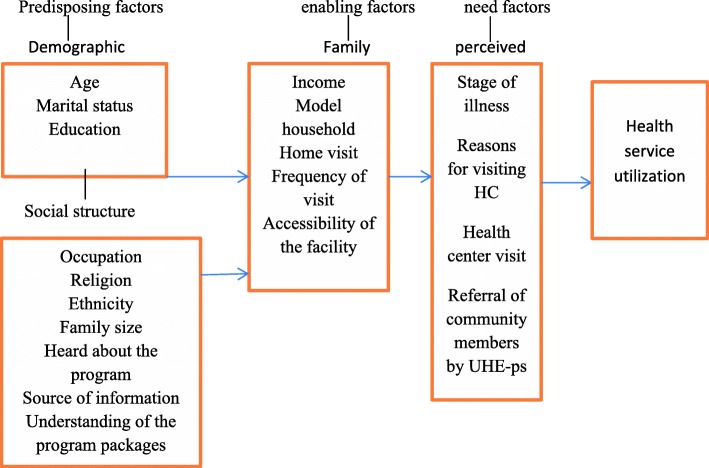
Anderson behavioral model of health service use

According to Anderson behavioral model risk factors associated to health service utilization can be categorized in to need, enabling and predisposing factors [6]. Health service characteristics can also have impacts on utilization of health services. These characteristics include the health service relevance (tertiary, secondary, or primary care); classification (drugs and medications, hospital, physicians and others); unit of measurements (episode, contact and volume); characteristics of provision, such as the intensity (amount) and time frame (acute or chronic); and frequency of household visits by the health workers [7, 8]**(**Fig. 1).

During the implementation period of the UHEP (particularly between 2012 and 2013 regarding child and maternal health services, an increase was observed for antenatal and postnatal care coverage (from 89.1 to 97.4% and from 44.5 to 50.5%, respectively) as well as for the percentage of deliveries attended by skilled health personnel (from 20.4 to 23.1%), while the percentage of clean and safe deliveries (by health extension workers) declined from 13.2 to 11.6% in the same period [9]. A report from a miniature demographic release in 2014 showed promising results in Addis Ababa, for example, in terms of utilization of any modern contraceptive method (57%), antenatal care (94), child mortality (53 per 1000 live births) and the rate of diarrhea in children under five (9.4%) [10].

This achievement logically links the launch of the UHEP to improvement of the health status of the urban community of Addis Ababa. However, investigation of the determinants and impacts of the program interventions would facilitate further progress toward sustaining development goals. Therefore, the aim of this study was to assess the utilization of the UHEP by the urban community and associated factors.

## Methods

### Study design and area

A community-based cross-sectional study was conducted among women in all 10 sub-cities of Addis Ababa in the month of January 2015 to assess health service utilization due to the UHEP and associated factors.

### Study population and sampling

The study population included women over the age of 18, since the majority of services provided by the UHEP involve women in reproductive age groups. The other reasons for focusing on women over 18 years old, aside from their position in the reproductive age group, were because they were actively involved in model household training and were more likely than individuals in the non-reproductive age group to finish the training, graduate and become certified [11].

A multistage sampling technique was used to select samples from 10 sub-cities (encompassing a number of woredas) of Addis Ababa. From all woredas (clusters) in each sub-city, a single woreda (cluster) was selected using a simple random sampling technique; from the selected woreda (smallest administrative unit with a population of 40,000), one ketena (the local catchment area within the subunit of the woreda) was selected using a simple random sampling technique; and from the selected ketena, the number of study units was specified based on the population in proportion to the sample size of the total population of the sub-city. The sampling flow was from the sub cities (the largest in terms of population) to the woredas (the components of the sub-city) to the ketena (the components of a woreda).

The unit of study for the quantitative part of this study was the household. To determine the number of households to be included in the study, a single population proportion formula for sample size calculation was used. There were 225,699 (29%) model households that had been trained and graduated in Addis Ababa out of the total 788,385 eligible households (Addis Ababa City Administration Health Bureau, Annual Performance Report 2014 unpublished**).** The number of model households is the basic indicator of the program and is used as a single proportion for sample size calculation.

*n* = (Z 1-α/2)^2^ p (1-p)/ d^2^).

*n* = (1.96)^2^ × 0.29x (1–0.29/0.04^2^.

*n* = 494 × 2(for the design effect) =988 + 10% non-response rate = 1086.

### Data collection

A structured questionnaire checklist was prepared based on the consultation of national and regional guidelines as well as previous health service utilization studies conducted in developing countries [6, 12]. The questionnaire was designed based on enabling, predisposing and need factors from the Anderson behavioral model of health service use. Face-to-face interviews of the female head (age over 18) of the sampled households were conducted using a structured questionnaire.

Twenty data collectors and 10 supervisors were recruited and participated in 5 days of training. The purpose of the training was to familiarize the survey team with the objectives of the study, procedures to be adhered to, and ethical issues. The data collectors were diploma-holding nurses, and the supervisors were BSc (Bachelor of Science) degree-holding nurse professionals and health officers who were familiar with the program.

**Pre-testing** checklists were applied in a pilot manner to 10% of the sample prior to the actual fieldwork. The quality of fieldwork during data collection has definite implications for the quality of the end result. Networks of enumerators, supervisors, technical working groups (TWGs), sub-city focal persons, woreda focal persons, local guides and principal investigators designed immediate solutions for the challenges faced during data collection. Supervisors were assigned and played roles in checking whether data collectors were collecting data as per the guidelines or not. They also checked the consistency and completeness of the collected quantitative data and submitted the data on the same day.

### Operational definitions

#### Model household

A household that implements 85% of the 15 different intervention health.

packages.

#### Non-model household

A household that implements less than 75% of the 15 different intervention health packages.

#### Voluntary community health workers

Community members who are at the frontline of the implementation of the program and volunteer to support UHE-ps.

#### Health development team leader

A community member who is at the frontline of the implementation of the program and implements 75% of the health packages.

#### Predisposing factors

UHEP-related (hearing of and understanding UHEP) and belief-related factors (perception of health services and health professionals) explaining an individual’s decision to use health services, any demographic parameter (sex, age, marital status), and social structure (education, occupation, family size, ethnicity, religion).

#### Enabling factors

Factors that influence utilization behavior (income, home visits by UHE-ps, frequency of home visits, services provided in the HC).

#### Need factors

Individuals’ perceived need to use heath care services based on their perceived illness and/or clinically evaluated illness (type and stage of illness).

#### Stable source of income

As mentioned, a persistent and consistent source of income.

#### Frequency of home visits by UHE-ps

The number of UHE-ps visits within 1 week or month, which we categorized as more-frequent visits if there was at least one visit per month and as no visits or less-frequent visits if there was no visit at all or if there was one visit within more than 1 month.

#### Health center

A government health facility serving the general public through the city administration of Addis Ababa.

#### Preference of health center

A patient’s or community member’s choice of health facility in the study area.

#### Health service utilization

Health center utilization by the community members in the study area.

The conduct of UHE-ps and the quality of services were assessed according to the study participants’ perceptions of UHE-p conduct and the services provided in the HC, respectively, using a 5-point Likert scale, ranging from 1 (poor) to 5 (very good). The comprehension/understanding of the UHEP was also assessed according to study participants’ perceptions of their UHEP knowledge using “Yes” or “No” responses, and study participants who mentioned the exact number of UHEP packages (i.e., 15 packages) were tagged as having “accurately remembered the number of UHEP packages”.

### Data analysis

Quantitative data were cleaned and entered into EPIDATA (created by EpiData Association, 1999, Denmark), and SPSS (Statistical Package for the Social Sciences, version17, SPSS Inc., 2007, Chicago) was used to analyze descriptive and inferential statistics.

#### Predisposing factor codes and definition

Age was coded as “0” for the non -reproductive age group (50–98 years) and “1” for the reproductive age group (18–49 years). Mothers’ occupations were coded as “0” for government-employed and other jobs “1” for housewives. Education was coded as “0” for illiterate and “1” for at least reading and writing. Hearing about the UHEP was coded as “0” for no and “1” for yes, and the understanding of the UHEP packages was coded as “0” for no and “1” for yes. Facility preference was coded as follows: 1 = health center (public), 2 = hospital (public), 3 = private (hospitals /clinics). “The ethno linguistic groups were labeled as follows: 1 = Amhara, 2 = Oromo, and 3 = others; religion was labeled as follows: 1 = Orthodox Christian, 2 = Muslim, 3 = Protestant”.

#### Enabling factors codes and definition

Model households were coded as “0” for no and “1” for yes. Home visits by UHE-ps were coded as “0” for no and “1” for yes, and the frequency of visits was coded as “0” for no visits (less-frequent visits) and “1” for frequent visits by UHE-ps (at least one visits every 4 weeks).

#### Need factors codes and definitions

Consultation of the UHE-ps in case of sickness was labeled as 1 = yes, and 2 = no. Reasons for visiting a health facility were labeled as follows: 1 = child illness, 2 = family planning, 3 = ANC, 4 = delivery, 5 = postnatal, 6 = family illness or 7 = immunization. The response to the question “At what stage of the illness do you visit the health facility?” was labeled as follows: 1 = soon after the illness starts, 2 = if there is no improvement, 3 = if the sick person is unable to eat /drink, or 4 = other.

Binary logistic regression was used to identify the predictors of HC visits by the community. Due to the multistage sampling procedure, individual women were nested within sub-cities; hence, the likelihood of women seeking HC visits was likely to correlate with sub-city residents. For the dependent variable of HC visits, two models were estimated: an intercept-only model, an empty model that contained no covariates, and a full model that included both individual variables and sub-cities. The intercept-only model allowed us to evaluate the extent of cluster variation in relation to the utilization of the UHEP.

Both the crude odds ratio (COR) and the adjusted odds ratio (AOR) were used for reporting the results of the binary logistic regression. The estimation of population parameters was presented with a 95% confidence interval (CI). To report statistical significance, a *P* value of 0.05 was taken as the cut-off point.

## Results

A total of 1086 households were sampled in the survey, and 1078 females participated in this study, corresponding to a response rate of 99.1%. Additionally, 954 of the study participants visited the health center during the year (89.1, 95% CI = 87.1, 90.9).

### Predisposing factors

The age of the respondents ranged from 18 to 98 years with a mean age of 39.14 ± 11.98 years. Most of the study participants (67.3, 95% CI = 64.5 to 70.2%) were married, (19.8, 95% CI = 17.5 to 22.2%) were illiterate, (64.3, 95% CI = 61.4 to 66.9%) and/or were housewives. Regarding religion and ethnicity, participants were mainly Orthodox Christian (77.3, 95% CI = 74.9 to 79.8), Amhara (44.7, 95%CI = 41.8 to 47.8%) and/or Oromo (24.4, 95%CI = 22.0 to 27%). The mean family size was 4.5 ± 2.25 individuals. Approximately 57.1% (95%CI = 54.0 to 60.0) of the participants had a sustainable source of income (Table 2).

**Table 2:** Socio-demographic characteristics of the study participants in Addis Ababa, Ethiopia, 2015 (*n* = 1078)

	Number (N)	Percent (%) (95% CI)
Variables		
Age group		
18–49	861	80 (77.8,82.4)
50–98	215	20 (17.6,22.2)
Total	1076	
Occupation		
Housewife	693	64.3 (61.4–66.9)
Government employed and other jobs	384	35.7 (33.1–38.6)
Total	1077	100
Steady source of income		
Yes	611	57.1 (54.1,59.8)
No	459	42.9 (40.2,45.9)
Marital status	1070	100
Married	723	67.3 (64.5,70.2)
Single, separated, widow or divorced	351	32.7 (29.8,35.5)
Total	1074	100
Total	1070	100
Number of family members		
2–6	853	84 (81.9–86.3)
7–11	156	15.4 (13.1–17.4)
12–16	7	0.7 (0.2–1.1)
Total	1016	100
Under-five children		
Have under-five children	707	65.6 (62.8–68.3)
Do not have under-five children	370	34.4 (31.7–37.2)
Total	1077	100
Religion		
Orthodox Christian	823	77.3 (74.9–79.8)
Muslim	162	15 (12.8–17.0)
Protestant	79	7.3 (5.8–9.1)
Total	1077	100
Educational status		
Illiterate	213	19.8 (17.5–22.2)
At least read and write	863	80.2 (77.8–82.5)
Total	1076	100

The majority (909; 84.4, 95% CI = 82.2 to 86.4%) of the study participants had heard about the UHEP, and the UHE-ps were the source of this information for 699 (76.9, 95% CI = 74.0–79.6%) of them. Out of the 1078 study participants, 571 felt that they understood/comprehended what the UHEP was.

Comparatively, the most frequently recalled packages were solid and liquid waste management, latrine construction and utilization, personal and environmental hygiene, and healthy housing, which were mentioned by 76.3, 74.2, 68.2, and 51.5% of study participants, respectively. The moderately recalled packages were tuberculosis, leprosy and HIV control, water supply and food hygiene, family planning, non-communicable diseases, and immunization, which were mentioned by 44.7, 36.7, 36, 29, and 24.2% of study participants, respectively. The least recalled packages were adolescent and reproductive health, health education, nutrition, first aid and accident prevention, which were mentioned by 17.9, 16.9, 15.5, 7.3 and 5.1% of the participants, respectively **(**Table 2). The conduct of the UHE-ps was rated as “good” by 93.0% (95% CI = 90.7 to 95.0%) of the participants. Similarly, the services provided in the HCs were viewed as being of “good quality” by 77.0% of participants (95% CI = 74.3 to 79.7) (Table 3).

**Table 3:** Knowledge and preferences of study participants regarding the Urban Health Extension Program in Addis Ababa, Ethiopia, 2015

Variables	Number (N)	Percent (%) (95% CI)
Community perception of health facility quality		
Good	740	77.0 (74.3–79.7)
Bad	221	23.0 (20.3–25.7)
Total	961	100
Community perception of the conduct of UHE-ps		
Good	541	93.0 (90.7–95.0)
Bad	41	7.0 (5.0–9.3)
Total	582	
Heard about UHEP		
Yes	909	84.4 (82–86.7)
No	168	15.6 (13.3–18.0)
Total	1077	100
Source of information		
UHE-ps	699	76.9 (74.0–79.6)
Other health professionals	43	4.7 (3.3–6.2)
Community members	77	8.5 (6.7–10.5)
Mass media	252	27.8 (24.9–30.8)
Tota**l**	1021	100
Understanding UHEP packages		
Yes	909	84.4 (82.3–86.6)
No	168	15.6 (13.4–17.7)
Total	1077	
Facility preference		
Health Center	880	81.8 (79.5–84.0)
Public hospital	34	3.2 (2.1–4.3)
Private hospital	158	14.7 (12.5–16.8)
Total	1072	
Which UHEP packages do you know of?		
Solid and liquid waste management	447	76.3 (72.9–79.7)
Latrine construction and utilization	435	74.2 (70.6–77.8)
Personal and environmental hygiene	399	68.1 (64.5–72.4)
Healthy housing	302	51.5 (47.4–55.5)
Tuberculosis, leprosy and HIV	260	44.7 (40.1–48.8)
Water supply and food hygiene	215	36.7 (32.9–40.4)
Family planning	211	36 (32.1–40.1)
Non-communicable diseases	170	29.0 (25.3–32.6)
Immunization	142	24.2 (20.8–27.8)
Adolescent and reproductive health	105	17.9 (14.8–21.2)
Health education	99	16.9 (13.8–20.0
Nutrition	91	15.5 (12.6–18.8)
First aid and accidental prevention	43	7.3 (5.3–9.6)
Malaria prevention and control	30	5.1 (3.4–7.0)
Total	1078	100

### Enabling factors

Three hundred eighteen (29.6, 95%CI = 26.9 to 32.3) households were model households; 614 study participants (57.1, 95% CI = 54.2 to 59.8%) received home visits by UHE-ps; and 391 (64.5, 95% CI = 60.4 to 68.3%) received frequent visits (at least one visits every 4 weeks). Most of the study participants (91.8, 95%CI = 90.0 to 93.6%) travelled on foot to visit the health centers (Table 4).

**Table 4:** Study participants’ income, social status, and Urban Health Extension Program-related status in Addis Ababa, Ethiopia, 2015

Variables	Number(N)	Percent (%) (95% CI)
Model household		
Yes	318	29.6 (26.9–32.3)
No	758	70.4 (67.7–73.1)
Total	1076	100
Sustainable source of income		
Yes	611	57.1 (54.0–60.0)
No	459	42.9 (40.0–46.0)
Total	1070	100
Home visits by UHE-ps		
Yes	614	57.1 (54.2–59.8)
No	461	42.9 (40.2–45.8)
Total	1075	100
Frequency of home visits by UHE-ps		
Frequent visits by UHE-ps (at least one visits every 4 weeks)	391	64.5 (60.4–68.3)
No visits (less frequent)	215	35.5 (31.7–39.6)
Total	606	
Means of transport to the health facility		
Own car	7	0.7 (0.2–1.2)
Public Transport	72	7.4 (5.8–9.1)
On foot	889	91.8 (90.0–93.6)
What UHE-ps do when they visit the home		
Teach us	482	78.1 (74.9–81.4)
Demonstrate to us	135	21.9 (18.6–25.1)
Total	617	100

### Need factors

The main reasons for visiting HCs were family illness, child illness and family planning, at 48.7% (95%CI = 41.3 to 56.1), 19.6% (95%CI = 14.3 to 25.4%) and 14.3% (95%CI = 9.5 to 19.6%), respectively. In cases of illness, 65.0% (95% CI = 62.0 to 67.8%) of the study participants visited the HC soon after the illness began, and 32.5% (95% CI = 29.7 to 35.4%) visited only if there was no improvement (Table 5).

**Table 5:** Reasons and stage of illness for seeking health facility visits and consultation with UHE-ps in Addis Ababa, Ethiopia, 2015

Variables	Number (N)	Percent (%) (95% CI)
Consultation of UHE-ps in case of sickness		
Yes	196	18.2 (16.1–20.5)
No	880	81.8 (79.5–83.9)
Total	1076	100
Reasons for visiting health facility		
Child illness	37	19.6 (14.3–25.4)
Family planning	27	14.3 (9.5–19.6)
ANC	6	3.2 (1.1–5.8)
Delivery	8	4.2 (1.6–7.4)
Postnatal	2	1.1 (0–2.6)
Family illness	92	48.7 (41.3–56.1
Immunization	4	2.1 (0.5–4.2)
Total	189	
At what stage of the illness do you visit the health facility?		
Soon after the illness starts	698	65.0 (62.0–67.8)
No improvement of the illness	349	32.5 (29.7–35.4)
If the sick person is unable to eat/drink	21	2.0 (1.1–2.8)
Others	6	0.6 (0.2–1.0)

### Association of Health Center Visits with household sociodemographic and health characteristics

The bivariate logistic regression showed that (HC) visits exhibited significant associations with mothers’ family incomes, hearing (having information) about the UHEP, model household status, households having under-five children, home visits made by UHE-ps and age categories. However, HC visits exhibited no significant association with mothers’ occupations, the frequency of visits, the understanding of the UHEP packages, the quality of health centers, or referral of community members by UHE-ps.

Mothers having a stable source of income were 0.63 times less likely to visit the HC **(**AOR = 0.63, 95%CI = 0.42 to 0.88) than mothers with no sustainable source of income. Mothers who had heard of or had information about the UHEP were 2.13 times more likely to visit the HC (AOR = 2.13, 95% CI = 1.36 to 3.32) than mothers who had not heard of the program. Mothers from model households were 2.12 times more likely to visit the HC (AOR = 2.12, 95% CI = 1.16 to 3.88) than mothers from non-model households. Mothers whose households were visited by UHE-ps were 1.89 more likely to visit the HC (AOR = 1.89, 95% CI = 1.22 to 2.94) than mothers whose households were not visited. Mothers who had under-five children were 1.96 times more likely to visit the HC (AOR = 1.96, 95%CI = 1.23 to 3.12) than mothers who did not have under-five children. Similarly, mothers who were in the reproductive age group (18 to 49 years) were 1.74 times more likely to visit the HC (AOR = 1.74, 95%CI = 1.12 to 2.71) than mothers older than 49 years. (Table 6).

**Table 6:** Factors associated with utilization of health services by households, Addis Ababa, Ethiopia, 2015 (*n* = 1078)

Variables	Utilization (Health Center visits) = D		Crude OR (95%CI)	Adjusted OR (95%CI)
	Yes	No		
Heard about UHEP				
Yes	820	83	2.57 (1.62,3.89)	2.13 (1.36,3.32) **
No	134	34	1.00	1.00
Home visit by UHE-ps				
Yes	569	42	2.61 (1.75,3.89)	1.89 (1.22,2.94) **
No	38	74	1.0	1.0
Sustainable source of income				
Yes	527	79	0.59 (0.38,0.88)	0.63 (0.42,0.88) **
No	427	37	1.0	1.0
Occupation				
Housewife	625	65	1.52 (1.03,2.24)	1.20 (0.76,1.88)
Govt employed and other jobs	325	52	1.0	1.0
Model household				
Yes	299	17	2.69 (1.58,4.58)	2.12 (1.16,3.88) **
No	654	100	1.0	1.0
Frequency of visit				
Frequent visits by UHE-ps (at least one visit every 4 weeks)	368	22	1.73 (0.92,3.26)	1.57 (0.82,3.02)
No visit (less frequent)	193	20	1.00	1.00
Households with under-five children				
Yes	611	313	2.07 (1.30,3.28)	1.95 (1.23,3.12) **
No	92	25	1.00	1.00
Referral of community members by UHE-ps				
Yes	88	865	3.87 (0.52,28.49)	3.44 (0.456,25.87)
No	1	38	1.0	1.0
Age category				
18–49	772	84	1.68 (1.09,2.59)	1.74 (1.12,2.71)**
50–98	181	33	1.0	1.0
Understanding the UHEP packages				
Yes	524	45	1.57 (0.99,2.48)	1.81 (0.71,4.65)
No	275	32	1.00	1.00
Community perception on HC quality				
Good	707	7	1.56 (1.17,2.08)	1.71 (1.19,2.47) **
Bad	245	6	1.00	1.00
Conduct of UHE-ps				
Good	499	40	17.69 (8.49,36.85)	20.39 (9.52,43.69)**
Bad	35	6	1.00	1.00

*D* Dependent variable

## Discussion

The results of showed being a model household, hearing (having information) about the UHEP, receiving home visits from UHE-ps, having under-five children, being a mother in the reproductive age group and having a sustainable source of income were independent determinants of HC utilization. However, HC visits exhibited no significant association with mothers’ occupations, the frequency of visits, understanding the UHEP packages, the quality of the health centers, or referrals of community members by UHE-ps.

Receiving household visits by UHE-ps and being model households exhibited significant associations with HC visits made by members of the community. The results of this study is in line with a study conducted in Ethiopia indicating that HEW (health extension worker) home visits and after becoming model households exhibited significant positive associations with health service utilization [13]. Other related study showed the potential of preventive home visits for reducing the disability burden, despite the fact that this depends on a number of risk factors and health facility infrastructure [14]. Another systematic review suggested that home visits can reduce chronic cares and mortality [15]. In contrast, a study by Van Haastregt and Diederiks (2000) showed that there was no any studies indicating the effectiveness of household visiting [16]. Another related study suggested that instant community involvements would have maximum benefit [17]. Nevertheless, some systematic reviews of home visiting programs have showed differences related to outcomes associated with the average intensities and durations of the programs [17, 18].

This study explored the hypothesis that model households increase the potential to utilize health centers. This hypothesis is in line with a related findings in Ethiopia showing that women who are model families of an HEP (health extension program) are more likely to exhibit good utilization of maternal health services [19].

Solid and liquid waste management, latrine construction and utilization, personal and environmental hygiene and healthy housing were the packages that were most frequently recalled. The moderately recalled packages included tuberculosis, leprosy and HIV control, water supply and food hygiene, family planning, non-communicable diseases and immunization. The least recalled packages were adolescent and reproductive health, health education, nutrition, first aid and accident prevention.

The packages that were least or moderately recalled could be due to the focus of UHE-ps on household-level services (even though the program implementation centers also include school and youth centers [1]), since most of the least-recalled packages were to be implemented among youths and adolescents. One exception to this trend was the malaria package, since the study area is not endemic for malaria.

In this study, mothers with a stable source of income were less likely to visit private or highly advanced centers (expensive centers) than mothers with no stable source of income. This discrepancy might have occurred because those mothers who had stable sources of income (affluent) were more likely to visit a government health facility (relatively cheaper centers), similar to mothers with no stable source of income, possibly due to the global and the local economic crisis in Addis Ababa, Ethiopia. This inference is consistent with a study from the USA indicating that hospitalization rates are higher in limited-resource areas than in higher-income areas, where appropriate outpatient care is more readily available [20]. However, this possibility is not consistent with studies from Uganda and Bangladesh that have reported income as one of the most important factors in overcoming barriers to the utilization of health services [8, 12, 21].

Mothers in the reproductive age group as well as mothers having children under five were more likely to visit the HC, possibly because most of the packages of the UHEP are related to this group of mothers and children under five. This study showed that HC visits exhibited no significant association with mothers’ occupations, the frequency of visits, the understanding of UHEP packages, the quality of health centers and referrals of community members by UHE-ps.

The findings of this study indicated that mothers’ occupations exhibited no significant association with HC visits, which is consistent with another related study conducted in Ethiopia showing that a mother’s occupation is not a predictor of health service utilization within the community of West Gojam, Ethiopia [13]. Similarly, a study performed in South Asia and sub-Saharan Africa indicated that the likelihood of using health care for maternal services are similar for both working and non-working women in Tanzania, Bangladesh, India and Pakistan [22]. Similarly, other related study in South Korean showed there is no differences in mortality rates due to occupational differences (between manual versus non-manual labour) [23].

However, studies conducted in Nepal indicated that women who are not working are more likely to utilize health services than working women, which is justified, since most women in Nepal work for family responsibility in the agriculture [24, 25]. A study from Iran also showed that housewives are more likely to utilize health services than other working women [26].

In the present study, numbers of household visits by UHE- ps displayed no significant association with HC visits. Contrary to this finding, a study from Nepal demonstrated that health worker visits showed as significant association with the health service use [27]. Awareness on the elements of the UHEP in this study is not predictor for the health service use. Another study from Ethiopia showed contrary findings, suggesting that awareness of the components of the packages was positively associated with the health service use [13]. Other similarly contrary findings from greater Lansing showed a statistically significant association between knowledge and level of literacy associated with the health service utilization [28]. However, results from a similar study in Atlanta indicated no association between the literacy level and health service uses [29].

Community perception toward the conduct of UHE-ps and services quality provided in the HC showed significant association with HC visits, which might be due to the expansion of health centers in the catchment areas of the urban community. However, contrary results from Ethiopia indicated that community attitudes towards ethical practices of the HEWs and services quality provided in the health facility displayed no significant association with health service utilization [13]. Opposing results from Uganda revealed that the community perceived governmental health facilities substandard cares [30]. Referral of community members by UHE-ps was not found to be a significant predictor of HC visits in the present study, most likely due to the attitude of the community and UHE-ps themselves, as the UHE-ps were entirely responsible for handling cases at the household level.

According to the Anderson behavioral model initially designed in this study based on predisposing factors (age, hearing about the UHEP, understanding the UHEP (comprehending what the UHEP was)) and enabling factors (model households, home visits by UHE-ps), these factors show significant associations with health service use, which implies that predisposing and enabling factors are well illustrated by this model. However, need factors were not well represented in the model.

## Limitations of the study

The UHEP related variable like home visit made by UHE-ps is the potential predictor in this study however similar related variable called frequency of visit by UHE-ps is not predictor variable for the health service utilization. Similarly hearing about UHEP (having information about the UHEP) is potential predictor, unfortunately understanding of the UHEP packages variable is not predictor for the health service utilization. This UHEP related variables expected to show causal ordering (but there is no casual ordering) so this needs further investigation.

The unique characteristics of this study included the use of a large sample size and a high response rate. Nevertheless, the two remaining limitations of this study were recall bias, the study participants might not have remembered all the UHEP services and associated factors, and a lack of baseline data with which to compare the findings of this study.

## Conclusions

The determinants of UHEP factors, such as being model households and receiving home visits from UHE-ps, exhibited significant positive associations with the health services program. Having a sustainable source of income also displayed a significant positive association with health service utilization. Sustaining practices of graduated and certified model households are essential to maximize the benefits of the UHEP activities for health service utilization. Home visits to both model and non-model households on a regular basis are essential to advance health service utilization, in addition to designing re-graduation or other sustainability options for those in graduated model households.

The UHE-ps have diplomas in clinical nursing and work in promotive and preventive aspects of the UHEP. The professionals’ career structures in their own field have not yet been established, which makes them unmotivated to continue as UHE-ps. Instead, most of them teach in private colleges with the aim of changing their profession. Designing UHEP-based undergraduate and higher educational training is of paramount importance to utilize the benefits of an innovative UHEP through minimizing the turnover of UHE-ps.

## Abbreviations

AA: Addis Ababa
AOR: Adjusted Odds Ratio
CI: Confidence Interval
COR: Crude Odds Ratio
DD: Dire Dawa
HC: Health Center
HDA: Health Development Army
HIV: Human Immunodeficiency Virus
HSU: Health Service Utilization
MH: Model Household
NMH: Non-Model Household
PMTCT: Prevention of Mother to Child Transmission
RRTEC: Research Review Technical Ethical Committee
SNNPR: Southern Nation, Nationalities, Peoples Region
SPSS: Statistical Package for Social Science
UHEP: Urban Health Extension Program
UHE-ps: Urban Health Extension professionals
